# Vitamin E Bioavailability: Mechanisms of Intestinal Absorption in the Spotlight

**DOI:** 10.3390/antiox6040095

**Published:** 2017-11-22

**Authors:** Emmanuelle Reboul

**Affiliations:** Aix Marseille University, INRA, INSERM, C2VN, 13005 Marseille, France; Emmanuelle.Reboul@univ-amu.fr; Tel.: +33-049-1324-278

**Keywords:** tocopherol, intestine, mixed micelles, dietary lipids, food matrix, membrane transporters, uptake, enterocytes, chylomicrons, HDL, fat-soluble vitamins

## Abstract

Vitamin E is an essential fat-soluble micronutrient whose effects on human health can be attributed to both antioxidant and non-antioxidant properties. A growing number of studies aim to promote vitamin E bioavailability in foods. It is thus of major interest to gain deeper insight into the mechanisms of vitamin E absorption, which remain only partly understood. It was long assumed that vitamin E was absorbed by passive diffusion, but recent data has shown that this process is actually far more complex than previously thought. This review describes the fate of vitamin E in the human gastrointestinal lumen during digestion and focuses on the proteins involved in the intestinal membrane and cellular transport of vitamin E across the enterocyte. Special attention is also given to the factors modulating both vitamin E micellarization and absorption. Although these latest results significantly improve our understanding of vitamin E intestinal absorption, further studies are still needed to decipher the molecular mechanisms driving this multifaceted process.

## 1. Introduction

**Figure 1:**
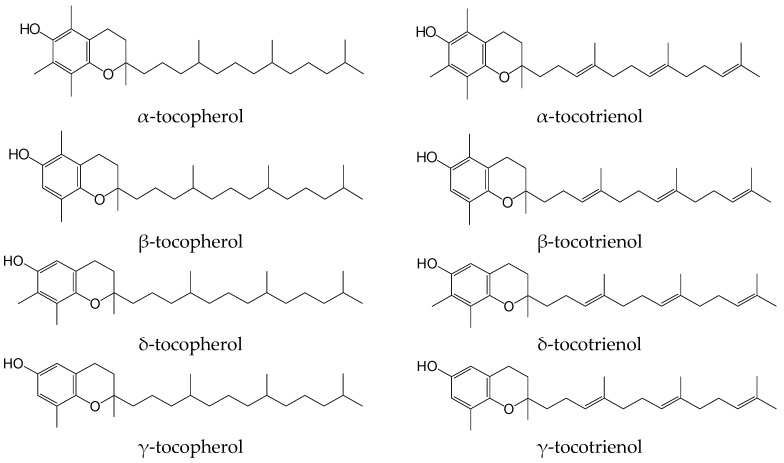
Vitamin E vitamers.

Tocochromanols, a subset of isoprenoids better known as vitamin E, include four tocopherols and four tocotrienols (Figure 1). These lipophilic antioxidants are synthesized by plants and other photosynthetic organisms only [1]. The base of the molecule of tocopherol is a hydroxychromane nucleus upon which a phytyl saturated chain of 16 carbon atoms is fixed. Three of these carbons are asymmetric, which entails the possibility of the existence of eight stereoisomers. The different tocopherols are distinguished from each other by the number and the position of the methyl groups attached to the nucleus. RRR-α-tocopherol is the most common in nature and the tocopherol with the highest biological activity. In biological tests for vitamin evaluation (fetal resorption tests), β and γ-tocopherol display a reduced vitamin activity (from 15 to 30%), and δ-tocopherol is almost inactive. Tocotrienols are distinguished from tocopherols by the presence of three double bonds on the side chain. Only α and β-tocotrienol appear to have a significant vitamin activity [2].

The hydroxychromane nucleus of vitamin E can react with peroxyl radicals, generating a hydroperoxide (which can be inactivated by specific enzymes) and a tocopheryl radical (which can be regenerated by vitamin C or coenzyme Q10). This property places vitamin E at the forefront of anti-radical defense systems [2]. However, the beneficial effects of vitamin E in human health may also be due to the ability of its phosphorylated metabolite to modulate signal transduction and gene expression in numerous conditions, including inflammation and immune system disorders [3].

The main sources of vitamin E are vegetable oils and seeds. It can also be found in smaller quantities in some fruits and vegetables (Table 1). In France and Europe in general, α-tocopherol is the most consumed vitamin E vitamer [2], while in the US, it is γ-tocopherol [4].

In Europe, the Recommended Dietary Allowance (RDA) for vitamin E is 11 mg per day (as an α-tocopherol-equivalent) for women and 13 mg per day for men [6], while the RDA is 15 mg for all adults in the US [7]. Although there are no real vitamin E deficiencies in Western countries, three surveys carried out in France (Burgundy, ESVITAF, and Val-de-Marne surveys) showed that more than 30% of French people consumed less than 8 mg of vitamin E a day [8], which was recently confirmed in other European countries [9] as well as in the US, where over 90% of the population do not consume the estimated average requirements [10]. As recently shown, the low dietary intake of vitamin E may be worsened by the low stability of vitamin E in vegetable oils [11].

## 2. Vitamin E Digestion Process

### 2.1. The Fate of Vitamin E in the Gastrointestinal Tract

The first phase of the digestion–absorption process is the dissolution of vitamin E in the lipid phase of the meal. This phase is then emulsified into lipid droplets at both gastric and duodenal levels. No metabolism of vitamin E (i.e., degradation or absorption) appears to exist in the stomach. In addition, the size of the droplets does not seem to have any effect on the efficiency of the subsequent absorption of the vitamin E in healthy humans [12].

In the duodenum, vitamin E is incorporated, along with lipid digestion products, in mixed micelles, structures that are theoretically essential for its absorption by the enterocyte. Indeed, mixed micelles can solubilize hydrophobic components and diffuse into the unstirred water layer (glycocalix) to approach the brush border membrane of the enterocytes.

### 2.2. Factors Affecting Vitamin E Transfer to Mixed Micelles

Numerous factors can affect vitamin E bioaccessibility (i.e., the fraction of vitamin E recovered in the mixed micelles compared to the initial amount of vitamin E provided by the meal) and thus in turn vitamin E bioavailability. The main factor is the food matrix in which vitamin E is embedded. For instance, it was shown that vitamin E bioaccessibility was low in apples but almost total in bananas, bread, or lettuce [13], and that vitamin E from durum wheat pasta was more bioaccessible than from pasta containing 10% eggs [14]. Unfortunately, it was not possible to identify the biochemical parameters of green leafy vegetables (cell-wall content, pectin, tannin, …) governing α-tocopherol bioaccessibility [15]. However, as for other lipid micronutrients, matrix disruption can enhance vitamin E transfer to mixed micelles [16], while thermal or high pressure treatments have either no or negative effects [17].

Another important factor determining vitamin E bioaccessibility is the amount of fat provided in the meal, as fat likely facilitates vitamin E extraction from its food matrix, stimulates biliary secretion, and promotes micelle formation. It was first shown that various fats and oils, as well as long-chain triacylglycerols, did not significantly enhance vitamin E bioaccessibility. This was in contrast to more hydrophobic microconstituents such as β-carotene [18]. However, further data consistently showed that tocopherol acetate bioaccessibility was higher in long-chain rather than medium-chain triglyceride emulsions, probably due to a greater solubilization capacity of mixed micelles formed from long chain fatty acids and an enhanced conversion into tocopherol [19,20,21]. Vitamin E bioaccessibility was also increased by the presence of phospholipids [22].

### 2.3. Vitamin E Ester Hydrolysis

It is acknowledged that only the free forms of vitamin E are absorbed by the intestinal mucosa, suggesting that the esterified forms are hydrolyzed beforehand. This hydrolysis is probably carried out by cholesteryl ester hydrolase, also known as bile salt-dependent lipase [23]. Conversely to what we observed for retinyl esters [24], neither pancreatic lipase nor pancreatic lipase-related protein 2 were able to hydrolyze tocopheryl esters [25]. Surprisingly, it has been recently reported that α-tocopherol acetate absorption was equivalent to that of free α-tocopherol in the absence of both digestive enzymes and bile salts in healthy subjects [26]. This result signifies that enzymes originating from the enterocytes, such as endoplasmic reticulum esterases [27], are able to realize this hydrolysis in a very efficient manner.

## 3. Vitamin E Absorption Mechanisms by the Enterocyte

### 3.1. Apical Transport at the Brush Border Level

When approaching the brush border membrane, mixed micelles are supposed to dissociate due to the existing pH gradient. The released constituents can then be captured by different more or less specific systems to be absorbed by the enterocyte. For more than 30 years, due to the first results obtained in rat intestinal everted sacs [28,29], vitamin E absorption has been considered to occur by passive diffusion through enterocyte apical membrane. However, we showed for the first time in 2006 that α- and γ-tocopherol absorption was mediated, at least partly, by scavenger receptor class B type I (SR-BI) [30]. It was also shown that NPC1 like intracellular cholesterol transporter 1 (NPC1L1) was involved in α-tocopherol [31,32] and γ-tocotrienol [33] absorption. We finally highlighted the additional role of CD36 molecule (CD36) in the tocopherol absorption process [34].

These three membrane proteins have primarily been described as cholesterol transporters in the intestine [35,36]. However, they do display a relatively broad substrate specificity. Besides cholesterol, SRBI can mediate carotenoid [37,38], vitamin D [39], and K [40] transport; NPC1L1 is involved in phytosterols [41], vitamin D [39], K [42] and lutein [43] uptake; and CD36 is involved in very long chain fatty acid [44], vitamin D [39], K [40] and carotenoid [37] absorption. It is thus not surprising that they are involved in vitamin E uptake as well. Both SR-BI [45] and NPC1L1 [46] were showed to traffic in clathrin-coated lipid vesicles after a lipid load. The fact that these transporters seem to selectively mediate the transport of some molecules present in mixed micelles is an argument in favor of a direct interaction with their ligands. Interestingly, it was recently demonstrated that α-tocopherol competed with cholesterol to bind to the NPC1L1-N terminal domain, promoting NPC1L1 endocytosis [47].

CD36 [48] and SR-BI [49] have recently been described as intestinal lipid sensors, and they appear to be key modulators of chylomicron secretion [50,51]. These roles suggest that their impact on vitamin E transport may actually be a consequence of their role in other lipid absorption process. Indeed, by promoting lipid fluxes through the enterocyte, these receptors would create a driving force for absorption of minor lipids such as micronutrients, due to the lipid gradient.

We observed that the presence of tocopherol in structures mimicking mixed micelles (i.e., containing a biliary salt and at least oleic acid) was necessary for transporter-dependent absorption in Caco-2 cells [32]. This is in agreement with another study in which we showed that both SR-BI and CD36 extracellular loops were able to bind postprandial mixed micelles in a more efficient manner than interprandial micelles [52]. However, this does not indicate whether this interaction is the first step of either a sensing or an absorptive process—or if the two phenomena are coexisting.

Finally, it is worth mentioning that we observed that vitamin E could be effluxed back to the lumen after being absorbed in Caco-2 cells. This efflux was SR-BI-dependent and was increased in the presence of acceptors, e.g., mixed micelles that did not contain vitamin E [30]. The in vivo relevance of such observation should be further investigated.

### 3.2. Vitamin E Trafficking across the Enterocyte

Once absorbed, the fate of vitamin E across the enterocyte has been poorly described. Being hydrophobic, vitamin E likely localizes into organelle membranes, cytosolic lipid droplets, or traffic bound to binding proteins. Subcellular localization revealed that vitamin E could accumulate in microsomal membranes, i.e., the endoplasmic reticulum, Golgi, lysosomal and peroxisomal membranes [53]. However, it should be noted that in this study, vitamin E was delivered to cells with Tween 40, which may have influenced its targeting within the cells compared to a delivery in its physiological vehicles (i.e., mixed micelles).

Targeting to microsomal membranes may occur either via clathrin-coated vesicles as mentioned above, or thanks to the intervention of cytosolic carriers. A tocopherol-associated protein (TAP) expressed in the intestine has been shown to bind vitamin E in human tissues [54], but it actually displays a weak affinity towards tocopherols [55]. Sec14p-like proteins TAP1, 2 and 3, also expressed in the human intestine, are probably better candidates as they improved tocopherol transport to mitochondria as efficiently as the α-tocopherol transport protein (α-TTP) [56]. Additional research is needed to definitely confirm their role in the enterocyte.

### 3.3. Basolateral Secretion to the Lymph or to the Blood Circulation

Most of the vitamin E is incorporated into chylomicrons in its free form at the Golgi apparatus level before being released to the lymph.

In mice, it has been showed that in addition to this apolipoprotein-B (apoB)-dependent route, an non-apoB pathway could exist [57]. This non-apoB route involves ATB-binding cassette A1 (ABCA1) that allows the secretion of vitamin E via intestinal High Density Lipoproteins (HDL) [58], and maybe the ATP binding cassette sub-family G member 1 ABCG1 [59,60]. However, this pathway seems to remain minor in humans. Indeed, mutations in microsomal triglyceride transfer protein (MTP) or in secretion-associated Ras related GTPase 1B (SAR1B), lead to abetalipoproteinemia and chylomicron retention diseases, respectively [61]. These pathologies, characterized by a lack of chylomicrons, are associated with a massive impairment of vitamin E absorption that is not balanced by another pathway [62,63].

### 3.4. Vitamin E Absorption Site in the Intestine

It has long been assumed that vitamin E, as with other lipids and lipid micronutrients, is absorbed in the upper half of the small intestine [64]. However, recent work from our laboratory highlighted that vitamin E absorption was in fact mainly located in the distal part of mouse small intestine, i.e., in the distal jejunum and the ileum [32,65]. This data seems conflicting with the fact that identified vitamin E intestinal transporters, i.e., scavenger receptors and NPC1L1, have been described as mainly expressed in the duodenum and the jejunum, respectively [36,66,67]. However, this can be partly explained by the subcellular localization of these proteins. For instance, SR-BI is mostly expressed at the apical side of the duodenal enterocytes, but it is present on the basolateral surface of the distal intestine [68]. Besides, a postmortem study in humans showed that the expression of CD36, NPC1L1, and ABCA1 was highly variable and displayed a bell-shape pattern, with the highest levels in the ileum [69].

### 3.5. Factors Modulating Vitamin E Absorption by the Intestinal Cell

As for vitamin E transfer to mixed micelles, numerous factors can influence vitamin E transport across the intestinal cell, which likely explains the important variations observed regarding vitamin E absorption efficiency. Indeed, different studies report efficiency in ranges of 10–95% [70,71,72]. However when deuterium-labeled vitamin E was used to assess absorption, this range was reduced to 10–33% [73].

The intestine does not seem to specifically discriminate between vitamin E stereoisomers [74], or between α- and γ-tocopherol [30,75]. However, a study found a higher absorption of α-tocopherol compared to γ- and δ-tocopherol in lymph-cannulated rats [76], which is consistent with the existence of a ω-hydroxylase that preferentially metabolized these two last vitamers in 3′ and 5′ carboxychromanol metabolites that can be excreted in the urine [77].

The food matrix can also influence specifically this step of vitamin E absorption. The presence of fibers did not modify vitamin E absorption in humans [78,79]. Conversely, lipids can be classified as effectors of vitamin E absorption as they can promote chylomicron formation. It is interesting to note that a minimal quantity of fat of 3 g was required for an optimal tocopherol absorption, and that increasing further this amount did not led to a better vitamin E bioavailability [80]. This was partly confirmed in another trial where α-tocopherol-acetate was almost negligible when ingested with 2.7 g fat [81]. However, these data are conflicting with other studies showing that the higher the amount of fat, the better vitamin E absorption [73,82], or conversely that dairy fat from whole milk does not increase vitamin E absorption compared to low-fat milk [83]. Mono and polyunsaturated fatty acid seem to promote vitamin E absorption compared to saturated fatty acids in cockerels [84] and in Caco-2 cells [85]. Conversely, phosphatidylcholine decreased α-tocopherol absorption efficiency in rats [86,87], an effect that was reversed by the presence of lysophosphatidylcholine [86]. Authors suggested that vitamin E was associated with phospholipids, leading to a low uptake. As we showed that the presence of phosphatidylcholine in mixed micelles was associated with a decreased binding of mixed micelles on scavenger receptor extracellular loops [52], we suggest that neutral phospholipids can also impact vitamin E absorption by modifying micellar interaction with the membrane proteins responsible for its uptake.

We showed that α-tocopherol E could compete for absorption with other lipid micronutrients such as γ-tocopherol and carotenoids [30,88], as well as vitamin A, D, and K [65] in Caco-2 cells. Except for vitamin A, these competitions are presumably due to common uptake pathways involving cholesterol transporters. Vitamin A uptake mechanisms are still unknown. However, it has been hypothesized that vitamin E was protecting vitamin A against oxidation in the intestine, leading to vitamin E degradation and reduced absorption in chickens [89]. Polyphenols such as naringenin could also reduce vitamin E uptake in Caco-2 cells [88]. The underlying mechanisms still need to be resolved, but we can suggest that polyphenols can impair (micro) nutrient absorption by interfering with membrane protein functioning, as previously shown with digestive enzymes [90]. Although this is still debated [91], a study showed that phytosterols (2.2 g per day during 1 week) could inhibit vitamin E absorption in normocholesterolemic subjects [92].

Finally, it is noteworthy that genetic factors including polymorphisms in genes coding for vitamin E and lipid intestinal metabolism such as SR-BI, CD36, ABCA1, ABCG1, or apoB have been associated with a modulation of vitamin E bioavailability in humans [93].

## 4. Conclusions

Overall, this review highlights the fact that the molecular mechanisms of both intraluminal fate and intestinal absorption of vitamin E are only partly understood to date. The discovery of vitamin E intestinal transporters with broad substrate specificity has raised many questions with respect to the potential interactions with dietary lipids during the vitamin E absorption process. Besides, it is likely that other proteins involved in vitamin E absorption still remain to be identified. The modulation of the activity of these proteins, including the existence of functional polymorphisms in their encoding genes and the regulation of their expression levels by epigenetic to post-translational factors, may explain much of the observed large interindividual variation in postprandial responses to vitamin E. Further dedicated investigations are needed to address these presumptions in order to offer adequate vitamin E-tailored recommendations to individuals.

## Figures and Tables

**Table 1:** Vitamin E food content [2,5]. Average values in brackets.

Foods	Vitamin E Content (mg/100 g)
Sunflower oil	0.1–90 (58.3)
Sunflower seeds	0.01–57.6 (42.3)
Other vegetal oils	0.1–30
Almonds	0.01–24 (14.6)
Butter	1.5–2.3 (2.11)
Fatty fish	0.9–2
Fruits and vegetables (spinach, tomatoes, etc.)	0.8–2
